# Surveillance of Antibiotic Resistance of Maltose-Negative *Staphylococcus aureus* in South African Dairy Herds

**DOI:** 10.3390/antibiotics9090616

**Published:** 2020-09-18

**Authors:** Joanne Karzis, Inge-Marié Petzer, Edward F. Donkin, Vinny Naidoo, Eric M.C. Etter

**Affiliations:** 1 Department of Production Animal Studies, Faculty of Veterinary Science, University of Pretoria, Private Bag X04, Onderstepoort 0110, South Africa; melklab@up.ac.za (I.-M.P.); eric.etter@cirad.fr (E.M.C.E.); 2 Department of Animal and Wildlife Sciences, University of Pretoria, Private Bag X20, Hatfield 0028, South Africa; este.vanmarle-koster@up.ac.za; 3 Department of Paraclinical Sciences, Faculty of Veterinary Science, University of Pretoria, Private Bag X04, Onderstepoort 0110, South Africa; christo.botha@up.ac.za; 4 CIRAD, UMR Animal, Santé, Territoires, Risque et Ecosystèmes (ASTRE), 34398 Montpellier, France; 5 ASTRE, University Montpellier, CIRAD, INRA, 34398 Montpellier, France

**Keywords:** antibiotic resistance, MIC, *Staphylococcus aureus*, mastitis, somatic cell count, seasons, regions

## Abstract

Antibiotic resistance has been reported since the 1940s in both human and veterinary medicine. Many years of monitoring milk samples in South Africa led to identification of a novel maltose-negative *Staphylococcus aureus* (*S. aureus)* strain, which appears to be an emerging pathogen. In this study, the susceptibility of this strain to antibiotics was evaluated over time, during diverse seasons in various provinces and according to somatic cell count (SCC) categories. A data set of 271 maltose-negative *S. aureus* isolates, from milk samples of 117 dairy herds, was examined using the disk diffusion method, between 2010 and 2017. This study also compared the susceptibility testing of 57 maltose-negative and 57 maltose-positive *S. aureus* isolated from 38 farms, from three provinces using minimum inhibitory concentration (MIC). The MIC results for the maltose-negative *S. aureus* isolates showed highest resistance to ampicillin (100%) and penicillin (47.4) and lowest resistance (1.8%) to azithromycin, ciprofloxacin and erythromycin. The maltose-negative *S. aureus* isolates showed overall significantly increased antibiotic resistance compared to the maltose-positive strains, as well as multidrug resistance. Producers and veterinarians should consider probability of cure of such organisms (seemingly non-chronic) when adapting management and treatment, preventing unnecessary culling.

## 1. Introduction

The genus Staphylococcus comprises various opportunistic pathogens of variable relevance in veterinary medicine. The most clinically relevant staphylococci in veterinary medicine are the coagulase-positive *Staphylococcus aureus* [1], members of the *S. intermedius* group [2], and the non-aureus staphylococci [3]. A noted property of staphylococci is their ability to develop resistance to antibiotics (for example by mutations). Methicillin resistance is of particular relevance because it is conferred by a presence of the *mecA* gene, encoding for producing an altered penicillin-binding protein (PBP) (*PBP2a* or *PBP2′*) with a low affinity for the beta-lactam antibiotics (penicillin, older cephalosporins and carbapenems) [4]. Methicillin-resistant *S. aureus* (MRSA) is recognised as problematic in human medicine. It is identified among the organisms causing the most challenging infections in hospitalised individuals and individuals in general [5].

In South Africa, *S. aureus* (maltose-positive) remains a challenge in udder health [6], even though this was reported as no longer the main challenge to udder health in most countries [7]. Mastitis caused by *S. aureus* remains a conundrum in South Africa because it is resistant to most antibiotics and cannot be cured. A high proportion of cows are culled for this reason, with an important negative economic impact on the dairy herd. The infected udder is considered the primary reservoir of *S. aureus*; the organism is believed to be transmitted during milking. Despite this, a proportion of heifers already infected with *S. aureus* enter the milking herd [8]. This suggests routes of transmission in addition to the milking equipment and the milking parlour. An adequate comprehension of *S. aureus* reservoirs and transmission is essential for the effective control of the organism in a herd. The expected treatment resulting in the cure of *S. aureus* (maltose-positive) infection can be estimated by considering the following factors; parity, the stage of lactation, the SCC level, the specific teat position on the udder and the number of quarters infected and the duration of the required treatment [9].

The milk laboratory at the Faculty of Veterinary Science at the University of Pretoria provided an extensive dairy cow udder monitoring programme in South Africa. Since 2005, an increasing number of coagulase-positive, maltose-negative staphylococci was isolated, confirmed as maltose-negative *S. aureus* by MALDI-TOF and 16S rRNA sequencing methods [10]. These organisms were first identified from a dairy cow in a single South African dairy herd with an average individual somatic cell count of less than 100,000 cell/mL of milk. Three years later, similar organisms were isolated from numerous dairy herds in South Africa, although with effective susceptibility to antibiotics assessed routinely, with a low SCC [11]. These organisms did not cause the same level of udder damage than maltose-positive *S. aureus* and did not cause any chronic or repeat cases in South Africa [11]. In more recent years (2016/2017/2018), individual coagulase-positive and maltose-negative staphylococci indicated resistance (MRSA, cefoxitin disk), and an increase in SCC of infected udders (> 400,000cells/mL milk) [11]. Inadequate informing concerning maltose-negative *S. aureus* from dairy cows also exists. A study in the Netherlands also isolated a maltose-negative strain of *S. aureus* [12]. The strain established in the Netherlands [12] differed from that investigated in this study in South Africa. In addition to the previous study [10], attempting to characterise this emerging pathogen, a further evaluation was conducted from the resistance trends, evident in historic disk diffusion susceptibility data, and more recent MIC data, the results of which are presented in this study.

The first and main objective of this study was to investigate the retrospective (disk diffusion) antibiotic surveillance data of unique maltose-negative *S. aureus* ST 2992 in various provinces, seasons and SCC categories over time. The second objective was to compare the MIC results of the maltose-negative *S. aureus,* previously identified as an emerging pathogen [10], to those of maltose-positive *S. aureus*. 

## 2. Results

### 2.1. Retrospective Data—Disk Diffusion Method

The first part of this study comprised the retrospective data analysis (disk diffusion) conducted on maltose-negative *S. aureus*, employing the latest CLSI breakpoints available during the study [13,14]. The eight antibiotics used in this retrospective study comprised the commonly used antibiotics available as intramammary remedies in South Africa. According to the univariate analyses, the proportion of resistance amongst the maltose-negative *S. aureus* isolates identified varied significantly per SCC category for cloxacillin. The proportions of resistance differed significantly between seasons for ampicillin, penicillin and cephalexin.

**Figure 1:**
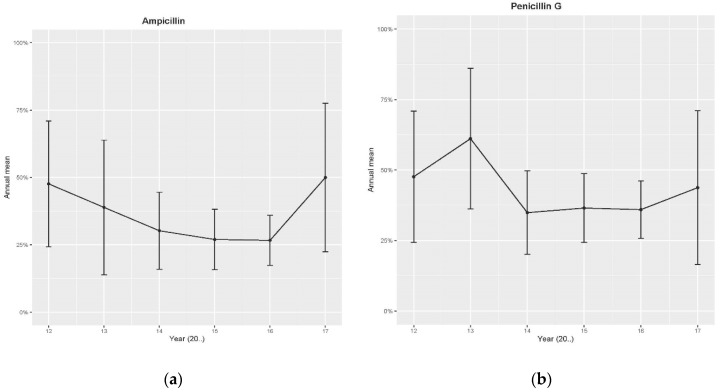

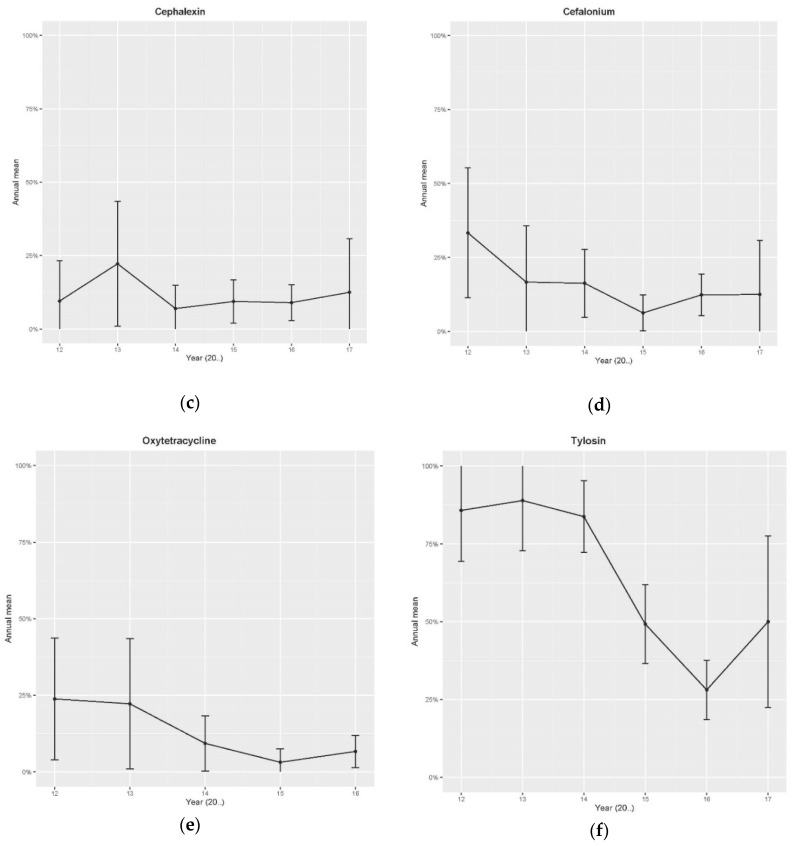
Trends of antibiotic resistance mean (proportion of isolates per year) of maltose-negative *S. aureus* (disk diffusion, retrospective data), according to CLSI [13,14]: (**a**) to ampicillin, (**b**) to penicillin G, (**c**) to cephalexin, (**d**) to cephalonium, (**e**) to oxytetracycline and (**f**) to tylosin.

The trends of penicillin G, ampicillin, cephalexin, cephalonium and oxytetracycline resistance peaked (at highest) in 2011, and for tylosin in 2013, then decreased over time (Figure 1a–f). In 2017, a slight increase in antibiotic resistance to ampicillin, penicillin G and tylosin but not for cephalexin, was indicated (Figure 1a–c,f). For Figure 1a–f, the represented period was limited to 2012 to 2017 as there were inadequate data for the previous years, resulting in exceptionally large confidence intervals.

The generalised linear mixed model (GLMM) results confirmed that time (years) was a significant variable for antibiotic resistance of maltose-negative *S. aureus* to penicillin G, ampicillin, cephalonium, oxytetracycline, cephalexin and tylosin. The association between the logit of resistance against tylosin and year was nonlinear (Table 1). According to the GLMM analysis, the SCC category represents the significant predictor for antibiotic resistance to cloxacillin (Table 1). The SCC category was significant for the cephalonium resistance (Table 1). “Season” was a significant variable for penicillin G, ampicillin and cephalexin. Spring was the season with the lowest level of resistance compared to autumn and summer for penicillin G and ampicillin, whereas autumn was the season with the lowest resistance level for cephalexin compared to summer. No effect of province on antibiotic resistance was detected (Table 1).

### 2.2. Minimum Inhibitory Concentration (MIC) Analysis

Table 2 displays the antibiotics used in this study, from the Pos MIC 32 panel (Beckman Coulter), indicating resistance to the isolates tested (Tables S1 and S2). The 57 maltose-positive and the 57 maltose-negative *S. aureus* isolates indicated resistance to ampicillin. From the total of 114 isolates, 37 were resistant to more than one antibiotic; 30 were maltose-negative *S. aureus* and seven maltose-positive *S. aureus*. A total of 25 multidrug-resistant (MDR) isolates (resistant to an antibiotic from three or more antibiotic categories) were indicated [15], of which three were maltose-positive and 22, maltose-negative *S. aureus* (Table S2). Multidrug resistance varied significantly between maltose-positive and maltose-negative *S. aureus* (*p* < 0.001).

## 3. Discussion

The breakpoints (CLSI) [13,14] are not just an indication of resistance, but they also consider concentrations at the udder level (i.e., its pharmacological resistance). In South Africa, a limited number of antibiotics are available as intramammary remedies, which consist mostly of ampicillin, cloxacillin or combinations thereof. The antibiotics with available breakpoints for mastitis specifically (ceftiofur, penicillin-novobiocin and pirlimycin), as indicated in the latest CLSI guidelines [13], are not available in the South African market. Other available CLSI veterinary breakpoints [13] were therefore used. For products on the Pos MIC 32 panel with no available veterinary breakpoints, the CLSI human breakpoints [14] were used, as explained and used in the publication which explains the development of veterinary antibiotic susceptibility guidelines over time [16].

This study indicated no significant differences (at a significance level of, *p* < 0.05) of antibiotic resistance amongst the provinces on the maltose-negative *S. aureus*, with a limited significant difference concerning seasons and SCC categories (Table 1).

According to the GLMM, the geographical origin (provinces) of the samples was not a significant predictor of the resistance (Table 1). A total of 271 isolates were identified, with an unequal distribution amongst the provinces. Despite the low number of isolates (<10) for six provinces, this indicated no difference in the resistance amongst Kwa Zulu Natal, Eastern Cape and Western Cape (with 170, 56 and 27 isolates, respectively). This discrepancy forbids a statistical analysis to demonstrate differences amongst all the provinces. The relationship of SCC to antibiotic resistance was similar to that of a study in Finland [17], which also established that in some cases a higher SCC could correspond with low antibiotic resistance and contrariwise. This could be attributable to the SCC being more of an indicator of irritation and severity of the infection rather than an indicator of antibiotic resistance of the organism. The study on maltose-positive *S. aureus* [18] indicated that the lowest prevalence of antibiotic resistance to most categories of antibiotics tested was present in KwaZulu-Natal during spring, except for cephalosporins, holding the lowest levels of prevalence of bacterial resistance in Gauteng during winter [18]. A possible reason for lower prevalence of antibiotic resistance of *S. aureus* to cephalosporins during winter in Gauteng may be that the dry cold season has generally a lower probable prevalence of intramammary infections that would require less treatment [18]. This would be supported by the occurring higher average incidence of frost duration in Gauteng [19], which would have suppressed insect vectors associated in mastitis pathogen transmission [20]. Reasons for these variations are unclear. Antibiotic resistance could also occur from random genetic mutations and subsequent natural selection of bacteria in order for them to survive [18].

Although great differences were identified in the numbers of herds and samples amongst the provinces, these differences were considered in the model during the analysis.

At that time (2009) a specific strain of maltose-negative *S. aureus* ST 2992 was identified [10], originating in and established mostly in KwaZulu-Natal, now established to be present in all provinces of South Africa, although in small quantities. The antibiotic resistance trends over time of this maltose-negative *S. aureus* (Figure 1a–f) agreed with those indicated for the same antibiotics with non-aureus staphylococci [21], but in contrast to the trends indicated for the maltose-positive *S. aureus* over time [22]. The maltose-positive *S. aureus* study indicated a general increase in resistance over time except for identified well-managed herds (part of the pro-active udder health programme) [22], indicating a decrease in resistance over time [22]. Other medical studies also showed a decrease in antibiotic resistance through the change in bacterial population resulting from good biosecurity measures [23]. In one medical study, a 9% year-on-year decrease in methicillin-resistant *S. aureus* (MRSA) cases was reported [24]. These medical studies have illustrated how the resistance profiles of bacteria can change under intensive care unit biosecurity programmes [23,24]. However, worldwide the increase of antibiotic resistance and rapid increase of resistant bacteria has reached a crisis, where many antibiotics which have transformed medicine and saved millions of lives are no longer effective against even the simplest infections [25,26]. Such infections often result in an increased number of hospitalisations, more treatment failures and the persistence of drug-resistant pathogens [25]. A recent World Health Organization (WHO) health report has warned that resistance to antibiotics in general is a “global” threat [27], and one that impacts on both human health care and the agricultural industry.

The MIC results (Table 2) confirmed the disk diffusion results (Table 1) for maltose-negative *S. aureus* for the tested products.

Table 2 summarises the distribution of the assessed antibiotics MIC test results for the respective maltose-positive and maltose-negative *S. aureus* isolates (Table S1). Twelve maltose-negative *S. aureus* isolates were resistant to oxacillin, compared to one maltose-positive *S. aureus*. The same pattern was observed for ertapenem; nine maltose-negative *S. aureus* isolates were resistant, compared to one maltose-positive *S. aureus*. The MICs of most products in the Pos MIC 32 panel differed between maltose-negative and maltose-positive *S. aureus* isolates (Table 2 and Table S1). The resistance rates of the maltose-positive *S. aureus* obtained in this study corresponded appropriately to those reported in additional studies [28].

The (MIC 50) represents the MIC value at which ≥50% of the isolates in a test population are inhibited, equivalent to the median MIC value. The (MIC 90) represents the MIC value at which >90% of the isolates in the test population are inhibited [29]. The MIC breakpoints are the chosen concentrations [μg/mL] of an antibiotic, defining whether a species of bacteria is described as susceptible or resistant to the antibiotic. Certain antibiotics were susceptible for MIC 50 and MIC 90 for both the maltose-negative and the maltose-positive *S. aureus*, except for MIC 90 of maltose-negative *S. aureus* (Table 2). The maltose-negative *S. aureus* was significantly more resistant (*p* < 0.001) to the amoxicillin–clavulanic acid combination (used in human medicine) and cefuroxime at MIC 90, and for clindamycin at MIC 50 (Table 2). Maltose-positive *S. aureus* was significantly more resistant (*p* < 0.001) to oxacillin for MIC 90 and MIC 50 and to clindamycin for MIC 90 (Table 2). Infrequently established resistance patterns were established in 18 of the 57 maltose-negative *S. aureus* isolates, resistant to vancomycin, and one maltose-positive and 12 maltose-negative *S. aureus* isolates were oxacillin resistant (Table S1) [30].

This study identified more multidrug-resistant maltose-negative *S. aureus* than maltose-positive *S. aureus* isolates (*p* < 0.001) (Table S2). The same interpretation applied for isolates resistant to two or more antibiotics of varying combinations. Several studies in animal and human medicine identified multidrug-resistant and pan drug-resistant *S. aureus* isolates [31]. Most of these studies were conducted on traditionally identified coagulase-positive, maltose-positive *S. aureus.* Although a coagulase-positive, maltose-negative *S. aureus* strain was subsequently isolated from bovine mastitis [12], no antibiotic susceptibility profiles of this organism were present. MALDI-TOF MS and 16S r RNA sequence analysis [10] identified the maltose-negative *S. aureus* ST 2992 isolated from milk samples in South Africa.

Human nasal *S. aureus* colonisation was previously reported as a pig farming risk factor [32]; *S. aureus* strains from pig farmers were present in pigs and not in non-farmers [32,33]. Considering pig research, it is possible that in the similar transmission of some strains of this resistant *S. aureus* (predominantly maltose-negative strains), isolated from dairy cattle in South Africa, could be from people. Previous studies in KwaZulu-Natal [34] identified Anthroponosis of *S. aureus* in South Africa, with one of the strains as the same maltose-negative *S. aureus* strain. Antibiotics approved for human use are a resource of unique antibiotics that should be kept for use in humans. Antibiotics approved for animal use only, such as the ionophores, should not create a risk to human health [35,36]. These maltose-negative *S. aureus* strains have different antibiotic resistance trends and antibiotic resistance severity compared to the traditionally identified maltose-positive *S. aureus* (Table 2). These two organisms react conversely in practice and should, therefore, be treated differently in practice [10].

This research identified 21 isolates of maltose-negative *S. aureus* with uncommon resistance profiles to antibiotics used in human medicine (such as the carbapenems; imipenem (*n* = 12) and ertapenem (*n* = 9)) [37]. These are antibiotics not used in animal medicine. Anthroponosis is a strong possibility because these isolates were present on the skin of humans who were in close contact with dairy cattle. This would be similar to the findings of the studies with the dogs in Brazil [38], pigs in Germany [32] and the Netherlands [39], respectively. Further studies are needed to explore the origin of such resistant isolates of maltose-negative *S. aureus*. Future work is also necessary to determine the resistance genes present in resistant maltose-negative *S. aureus* strains.

A similar study in dairy cattle in Tennessee also established a variation of prevalence of antibiotic resistance of *S. aureus,* with an increasing trend in tetracycline resistance [40]. These conclusions followed the report identifying the predominant antibiotic groups used in animal health in South Africa from 2014 to 2015, as the growth promoters (animal use only antibiotics) (62%) followed by tetracyclines (17%) and macrolides (11%) [41]. This study indicated five isolates resistant to tetracyclines, and ten isolates resistant to macrolides. Only one of each isolate was maltose-positive, whereas the rest were maltose-negative *S. aureus*.

Antibiotics used in South Africa in 2015 were identified as 21,149 standard units per 1000 human population (IMS Health 2015) (Note: 1 standard unit is equivalent to one tablet, or injection); this was significantly higher than in most other countries globally. Broad-spectrum penicillin use in humans in South Africa was 1.3 to 3.3 times higher than in other countries and 0.8 times higher than in the United Kingdom or the USA [41]. It is undecided if the high number of maltose-negative *S. aureus* isolates resistant to penicillin and ampicillin might solely be attributed to the high antibiotic usage.

The antibiotic resistance trends (Figure 1a–f) and profiles (Table 2), allow for informed treatment decisions without pausing for specific antibiotic sensitivity test results where animal-specific breakpoints are used [16]. The antibiotic sensitivity test results of (disk diffusion or MIC), are an indication in vitro that the particular organism is liable to be destroyed by a particular antibiotic. In the udder, the situation may be different because the site of the infection may be difficult to reach through small arteries and lactiferous ducts, attributable to udder pharmacokinetics (very few products are successful in a water and fat environment). As a result, mastitis treatment success is not exceedingly high (27%) [42] and can lead to antibiotic resistance development by mastitis-causing organisms. Although treatment against *S. aureus* is generally more successful in the dry period, it is still not ideal [43]. Therefore for mastitis control, the focus should be on the proactive udder health programme prevention and monitoring [44], rather than on treatment alone.

## 4. Materials and Methods

The milk laboratory of the Faculty of Veterinary Science (University of Pretoria, Pretoria, Gauteng, South Africa) received samples from most commercial dairies in South Africa since 1999, as part of the proactive udder health programme (routine testing of microbiology and cytology, every two to three months) [22]. This sampling entailed mostly routine whole herd investigations, which included all the lactating animals in the herd and detecting all intramammary infections, clinical and subclinical. Bacteriological analyses are conducted routinely on these samples. Two-hundred-and-seventy-one maltose-negative *S. aureus* isolates were identified from 117 dairy herds from the samples received from 2000 dairy farms between 2009 and 2017 [45], with 4, 8, 4, 21, 18, 44, 65, 91 and 16 isolates, for the years 2009, 2010, 2011, 2012, 2013, 2014, 2015, 2016 and 2017, respectively. The milking intervals for these dairy herds were either 12 or 8 hourly, depending on the number of milkings per day. Each herd was approached on an individual basis. The lactating cows in the 117 herds in this study varied from 30 (smallest herd) to 1700 cows (largest herd) [45]. The study population included mainly Holstein Friesian, Holstein crossbreeds, crossbreeds and Jersey dairy cows. Cows differed in age, parity, days in milk and milk yield. These herds were located in the nine provinces of South Africa, with unbalanced numbers of organisms among the provinces, indicating, Gauteng (*n* = 8), KwaZulu-Natal (*n* = 170), Free State (*n* = 4), Eastern Cape (*n* = 56), Western Cape (*n* = 27), Northern Cape (*n* = 1), North West (*n* = 1), Limpopo (North) (*n* = 1) and Mpumalanga (*n* = 3). The maltose-negative *S. aureus* isolated per farm varied from one to nineteen.

The single foremilk milk samples were collected in an aseptic manner according to a standard operating procedure [46]. Sampling was performed in the parlour prior to milking, at the routine milking intervals of each farm, as described above. Milk samples were taken by professional samplers or milkers trained according to a standard operating procedure [46]. Prior to sampling, the first milk was stripped from all quarters and the teat ends were carefully cleaned and disinfected with methylated alcohol. Approximately 10 mL of milk was collected in an aseptic manner into sterile marked sample tubes and kept refrigerated until shipment. In the case of composite milk samples, the same procedure was followed, but approximately equal volumes of milk from each of the four quarters were collected in one sample tube [46]. Milk samples were submitted by producers, veterinarians and field workers within 48 h after sample collection on ice to the laboratory [22]. Temperatures and conditions such as sample tube cleanliness and appearance were noted on arrival at the laboratory, and samples that were spoiled or of doubtful quality were not processed. Samples were plated out at the laboratory on the day of their arrival. Most of these dairy producers send composite milk samples to the Milk Laboratory, Faculty of Veterinary Science, University of Pretoria, for testing (microbiology and cytology) on a routine basis, as part of a proactive udder health management programme. In the case of mastitis outbreaks or clinical mastitis cases, quarter milk samples are used as a follow-up test method [22]. These isolates were collected and cultured according to the method recommended by the National Mastitis Council [46]. The laboratory investigation took place in the milk laboratory at Department of Production Animal Studies, Faculty of Veterinary Science, University of Pretoria. All milk samples were visually inspected and were then cultured on bovine blood tryptose agar (BTA) (Columbia Blood Agar Base, CM331 from Oxoid, plus 5% defibrinated bovine blood) and were incubated at 37 ± 1 °C for 24 to 48 h [46]. All samples in the current data set were diagnosed by using one or more colonies in cases where *S. aureus* was suspected and two or more in all other cases. Only pure cultures were used. Colonies were initially identified based on colony morphology, haemolysis and potassium hydroxide (KOH) test results [47]. The catalase reaction was used to differentiate between Gram-positive staphylococci and streptococci. Staphylase™, a coagulase test (Oxoid, supplied by Quantum Biotechnologies (Pty) Ltd., Ferndale, South Africa), was used to distinguish between coagulase-positive and coagulase-negative staphylococci. Maltose agar plates (Merck NT Laboratory Supplies, Halfway House, South Africa) were used for further identification of staphylococci. A positive maltose agar reaction confirmed *S. aureus*; a negative maltose reaction confirmed an organism potentially from the *S. intermedius* group [30,41], on initial phenotypic identification [10]. These organisms were identified using the MALDI-TOF and 16S sequencing. Both these methods confirmed a maltose-negative strain of *S. aureus* [10]. Further multi-locus sequence typing (MLST) and analysis of the *MalA* and *MalR* genes, revealed maltose-negative *S. aureus* ST 2992 with an abnormal stop codon on the *MalA* gene (GenBank accession number, MN531305) [10]. Microbiological and cytological examinations were performed on all milk samples [46].

Somatic cell counts were counted by fluoro-opto-electronics, using a Fossomatic 5000 and Fossomatic FC (Rhine Ruhr). Isolates to be tested for routine antibiotic susceptibility were selected from milk samples with a somatic cell count (SCC) (Fossomatic 5000 and Fossomatic FC, Rhine Ruhr) of over 400,000 cells/mL [8,21], when applicable, to include subclinical mastitis cases. This was the general rule for routine antibiotic susceptibility testing. When a maltose-negative *S. aureus* was isolated from a herd, antibiotic sensitivity testing was conducted on that organism, regardless of SCC [10].

The Kirby–Bauer disk diffusion method [48] was used to determine the antibiotic susceptibility of the routine diagnostic samples for the retrospective data analysis. At least four to five well-isolated colonies of the same morphological type from an agar plate were selected for the Kirby–Bauer disk diffusion test [48]. The results were based on the inhibition zones diameter and were classified as sensitive, intermediate or resistant under the latest clinical breakpoints available during the study, CLSI [13,14].

Antibiotic susceptibility was tested against nine intramammary antibiotics (in lactation and dry cow therapy) available in South Africa. These were the penicillin (ampicillin 10 μg, cloxacillin 5 μg, penicillin G 10 IU), cephalosporins (cephalexin 30 μg, cefuroxime 30 μg, cefoxitin 30 μg), lincosamides (clindamycin 10 μg), tetracyclines (oxytetracycline 30 μg) and macrolides (tylosin 30 μg).

Minimum inhibitory concentrations tests were conducted on 57 coagulase-positive, maltose-negative *S. aureus* isolates (2012–2013 *n* = 15; 2018–2019 *n* = 42) and 57 maltose-positive *S. aureus* isolates (2012–2014 *n* = 11; 2017–2018 *n* = 46), isolated from 38 dairy herds mainly from KwaZulu-Natal and the Eastern Cape, with a few samples from the Western Cape, Gauteng and Mpumalanga. The 57 maltose-negative *S. aureus* isolates were the total number of these isolates collected during the mentioned periods. For the maltose-positive *S. aureus* isolates, the same number (*n* = 57) of isolates from the same farms, with similar corresponding SCC ranges and from similar periods to those of the maltose-negative *S. aureus* samples were selected. The selected antibiotic agents were based upon the availability of commercial intramammary infusion products or as representatives of their respective antibiotic classes, such as ampicillin, oxacillin, erythromycin, penicillin and tetracycline.

The MIC was determined by employing the automated broth microdilution method (Pos MIC 32 panels and Microscan 40 Walkaway system, Beckman Coulter, Brea, CA, USA). The antibiotics and concentrations used for the MIC testing were as per the specifications on the package insert of the commercially available PM 32 panel (Table 2). The results were evaluated according to CLSI [13,14]. *Staphylococcus aureus* ATCC 25923 and *S. pseudintermedius* ATCC 49444 functioned as reference strains for quality control purposes. The MIC data analysis used the LabPro software of the Microscan 40 Walkaway system (Beckman Coulter, Brea, CA, USA) to determine the MIC 90; the MIC 50 was calculated manually.

To analyse various factors that could affect the resistance of the isolates, a two-step approach was used. Univariable analyses were first applied to explore the potential effects of these factors. Univariate multivariable analyses [49] were then used to confirm these effects and to explore interactions.

The Chi-square test was used to verify the existence of any effect of season or SCC category on the response variable, including the resistance to the various antibiotics. The used SCC categories were low (< 150 × 10^3^ cells per ml milk), medium (150 × 10^3^ to 400 × 10^3^ cells per ml milk), and high (> 400 × 10^3^ cells per ml milk). The seasons were defined as spring (September to November), summer (December to February), autumn (March to May) and winter (June to August). The Fisher exact test was applied to all nine provinces, testing this geographical effect attributable to the small numbers of isolates for six of the provinces.

These Chi-square and Fisher tests also allowed classification of the categories of each variable to introduce the variable with the lowest level of resistance as the reference category in the following generalised mixed model (GLMM) analysis. Generalised linear mixed models are models considering non-independence amongst clustered observations. The random variable defines the clusters. As generalised models, they allow a nonlinear relationship between the dependent variable (variable to be explained) and the model parameters [50]. In the univariate multivariable GLMM [40], the drug-specific antibiotic resistance of all *S. aureus* isolates was used as the dependent variable. As it is a binomial variable a logit link function was used. The herd was introduced as the random variable. Then, season, province and SCC category were introduced as different categorical independent variables (model parameters). The year was added as a numeric independent variable. The quadratic and cubic effect of year was tested for each antibiotic, which indicated an effect of the year.

Under the “goal of parsimony”, a stepwise approach based on the smallest Akaike Information Criterion was used to select the worthiest model [51]. The likelihood ratio test was applied to test the significance of the retained fixed effects. *P*-values lower than 0.05 were considered as statistically significant; *p*-values between 0.1 and 0.05 were considered as almost significant. The analysis was conducted separately for each antibiotic, employing the R software © version 3.3.3 “lme4” and “afex”.

The Fisher’s exact test was conducted to compare the resistance level of maltose-negative and maltose-positive *S. aureus* for each antibiotic and multidrug resistance.

The study presents a retrospective analysis approved by the University of Pretoria Ethics Committee (reference number V062/14). The laboratory supplying the data provided a written consent from owners for data to be used for research purposes, approved by the ethics committee.

## 5. Conclusions

The antibiotic resistance inclinations for ampicillin, cephalexin, cephalonium, cloxacillin, oxytetracycline and penicillin G peaked (at highest) in 2011 and for tylosin in 2013, subsequently decreasing. These antibiotic resistance trends over time revealed a closer comparison with the analysis of similar data for non-aureus staphylococci than for the maltose-positive *S. aureus*. Antibiotic resistance trends over time also differed between maltose-negative and maltose-positive *S. aureus*. Antibiotic resistance of maltose-negative *S. aureus* indicated no significant differences amongst provinces, with limited differences amongst seasons for ampicillin, penicillin G and cephalexin. For cloxacillin and cephalonium specifically, high SCC corresponded with low antibiotic resistance, significantly different from low SCC to correspond with a high antibiotic resistance cephalonium. The MIC antibiotic resistance results for maltose-negative *S. aureus* confirmed the disk diffusion method results. This showed that the Kirby–Bauer method, in addition to being a relatively quick and cost-effective method, is also accurate enough for routine veterinary diagnostics for antibiotic sensitivity testing in dairy cows. The results of the MIC method also indicated more resistance for maltose-negative than for the maltose-positive *S. aureus* isolates to most of the antibiotics used. The MIC breakpoints were susceptible at MIC 50 and MIC 90 for maltose-negative and maltose-positive *S. aureus*, except for maltose-negative *S. aureus* at MIC 90. These findings also showed resistance of the maltose-negative strain isolated from milk samples to antibiotics that are only used in human medicine, which implies a possible anthroponosis (transfer from humans to animals) and requires further studies under the “one health” approach. This study also highlighted the differences in antibiotic resistance profiles between the maltose-positive and maltose-negative *S. aureus*. This is useful information for both producers and veterinarians, in order to adapt different management and treatment protocols for this maltose-negative *S. aureus* which seems not to be a chronic intra-mammary infection, preventing unnecessary culling.

This highlights the importance of individual organisms in antimicrobial resistance which is shown by the higher antimicrobial resistance of maltose-negative *S. aureus* ST 2992 compared to that of maltose-positive *S. aureus*, as well as multidrug resistance. The results of this surveillance study and the availability of antibiotic resistance profiles for the maltose-negative *S. aureus* will allow for informed decisions to be made on treatment and management of this emerging pathogen in practice.

## Figures and Tables

**Table 1:** Summary variables on antibiotic resistance effects of GLMM for maltose-negative *S. aureus*, according to the CLSI breakpoints used [13].

Antibiotics	Year	Season	Province	SCC Category
Tylosin	Cubic effect (*p* < 0.001 for Year, Year^2^ and Year^3^)	NS	NS	NS
Penicillin G	*p* = 0.018	*p* = 0.05 [Autumn *p* = 0.03 & Summer p = 0.02] vs. Spring (Lowest R)	NS	NS
Ampicillin	*p* = 0.008	N/A	NS	NS
Clindamycin	N/A	NS	N/A	N/A
Cefuroxime	NS	NS	NS	NS
Cephalonium (2011–2017)	*p* = 0.03 *	NS	NS	*p* = 0.027 [High SCC *p* = 0.007] vs. Med SCC (Lowest R)
Cefoxitin (2014–2017)	NS	NS	NS	NS
Oxytetracycline	*p* < 0.001	NS	NS	NS
Cephalexin	*p* = 0.017	*p* = 0.06 [*p* = 0.01 Summer] vs. Autumn (Lowest R)	NS	NS
Cloxacillin	NS	NS	NS	*p* = 0.022 [Low SCC *p* = 0.08] vs. High SCC (Lowest R)

* Almost significant *p* < 0.1, NS = Not Significant, R = Resistance, N/A = Not applicable.

**Table 2:** The minimum inhibitory concentrations (MIC) 90 and MIC 50 for maltose-positive and maltose-negative S. aureus.

Product	% Resistance Maltose-Positive *S. aureus* (*n*)	% Resistance Maltose-Negative *S. aureus* (*n*)	MIC 90		MIC 50		Fisher’s Exact Test *p*-Value
			Maltose-Positive *S. aureus*	Maltose-Negative *S. aureus*	Maltose-Positive *S. aureus*	Maltose-Negative *S. aureus*	
Amox/K Clav	3.5 (2/57)	21.1 (12/57)	1	2	0.5	0.5	0.008 **
Ampicillin	100 (57/57)	100 (57/57)	4	4	0.5	0.5	
Azithromycin	3.5 (2/57)	1.8 (1/57)	1	1	_	_	
Cefepime	1.8 (1/57)	22.8 (13/57)	4	4	_	_	
Cefotaxime	1.8 (1/57)	22.8 (13/57)	1	2	_	_	*p* < 0.001 **
Cefoxitin	1.8 (1/57)	0 (0/57)	4	4	_	_	
Cefuroxime	1.8 (1/57)	21.1 (12/57)	4	8	_	_	0.002 **
Ciprofloxacin	7 (4/57)	1.8 (1/57)	1	0.5	0.5	_	
Clindamycin	5.3 (3/57)	24.6 (14/57)	0.3	_	_	0.25	0.007 **
Daptomycin	0 (0/57)	17.5 (10/57)	0.5	_	_	0.5	0.001 **
Ertapenem	1.8 (1/57)	15.8 (9/57)	0.5	0.5	_	0.5	0.016 *
Erythromycin	3.5 (2/57)	1.8 (1/57)	1	_	_	1	
Fosfomycin	1.8 (1/57)	12.3 (7/57)	32	_	_	32	
Fusidic Acid	0 (0/57)	10.5 (6/57)	2	_	_	2	0.027 *
Gentamicin	7 (4/57)	22.8 (13/57)	1	2	_	1	0.033 *
Imipenem	3.5 (2/57)	21.1 (12/57)	2	2	_	_	
Levofloxacin	0 (0/57)	0 (0/57)	1	1	_	_	
Linezolid	0 (0/57)	15.8 (9/57)	2	_	1	2	0.003 *
Meropenem	1.8 (1/57)	21.1 (12/57)	2	2	_	_	
Moxifloxacin	0 (0/57)	0 (0/57)	0.5	0.5	_	_	
Nitrofurantoin	0 (0/57)	8.8 (5/57)	64	64	_	_	
Oxacillin	1.8 (1/57)	21.1 (12/57)	0.3	_	0.5	0.25	0.002 **
Penicillin	36.8 (21/57)	47.4 (27/57)	_	_	0.03	0.12	
Rifampin	0 (0/57)	5.3 (3/57)	0.5	0.5	_	_	
Synercid	5.3 (3/57)	19.3 (11/57)	1	_		1	0.043 *
Teicoplanin	0 (0/57)	14 (8/57)	1	_	_	1	0.006 **
Tetracycline	7 (4/57)	7 (4/57)	1	1	1	_	
Tobramycin	3.5 (2/57)	7 (4/57)	1	1	_	_	
Trimeth/Sulpha	0 (0/57)	0 (0/57)	1	1	_	_	
Vancomycin	0 (0/57)	31.6 (18/57)	2	_	1	1	

Amox/K Clav = Amoxicillin Clavulanate; Trimeth/Sulpha = Trimethoprim/Sulphamethoxazole; *n* = number of isolates; * significant *p* < 0.05; ** significant *p* < 0.001.

## Supplementary Materials

The following are available online at https://www.mdpi.com/2079-6382/9/9/616/s1, Table S1: Distribution of minimum inhibitory concentrations (MIC) cumulative percentage for maltose-positive and maltose-negative *S. aureus*, Table S2: Minimum inhibitory concentration (MIC) results of maltose-positive and maltose-negative *S. aureus* resistant to more than one antibiotic.
