# Supplementary material for: Surveillance of Antibiotic Resistance of Maltose-Negative Staphylococcus aureus in South African Dairy Herds
Source: Antibiotics (Basel). 2020 Sep 18;9(9):616. doi: 10.3390/antibiotics9090616 (PMC7559053; doi:10.3390/antibiotics9090616)
Supplement: Supplementary file 1 [file antibiotics-09-00616-s001.pdf]

Table S1. Distribution of minimum inhibitory concentrations (MIC) cumulative percentage for maltose positive and maltose negative *S. aureus*.

Product	% Resistance	STA (+) (n = 57) Distribution of MICs % (μg/ml)												% Resistance	STA (-) (n = 57) Distribution of MICs % (μg/ml)										
		0.03	0.06	0.12	0.25	0.5	1	2	4	8	32	64	256		0.03	0.06	0.12	0.25	0.5	1	2	4	8	32	64
Amox/K Clav	3.5					89	99	99	99	99				21.1					77	89	91	95	100		
Ampicillin						70	78	85	90	90									56	63	68	93	96		
Azithromycin	3.5						98	98						1.8						98	98				
Cefepime	1.8								100	100				22.8								98	98		
Cefotaxime	1.8						93	100						22.8						88	96				
Cefoxitin	1.8								100					0								100			
Cefuroxime	1.8								100	100				21.1								89	96		
Ciprofloxacin	7					89	94							1.8					95	98					
Clindamycin	5.3				91	96	_	98						24.6				68	77	_	86				
Daptomycin	0					95	100	100	100					17.5					82	82	82	82			
Ertapenem	3.5					98*	98				100			1.8					97	97					
Erythromycin	1.8						99	99						15.8						82	84				
Fosfomycin	1.8										98			12.3										88	
Fusidic Acid	0							100						10.5							89				
Gentamicin	7						93	98	100					22.8						79	98	98			
Imipenem	3.5							99	99	99				21.1							100	100	100		
Levofloxacin	0						100	100						0						100	100				
Linezolid	0					21	64	100	100					15.8					21	37	82	84			
Meropenem	1.8							100	100	100				21.1							100	100	100		
Moxifloxacin	0					100	100*							0					100	100					
Nitrofuratoin	0											100		0											91
Oxacillin	1.8				54	91	98	100						21.1				56	79	88	88				
Penicillin	36.8	61	70	70*	70	_	_	76						47.4	33	40	53	54	_	_	63				
Rifampin	0					100	100	100						5.3					93	95	95				
Synercid	5.3						100	100	100					19.3						75	77	82			
Teicoplanin	0						100	100	100	100				14						82	84	8	86		
Tetracycline	7						92	92						7						95	95				
Tobramycin	3.5						98	98	100					7						93	98	98			
Trimeth/Sulfa	0						100	100	100					0						98	98	100			
Vancomycin	0				0	27	86	100	100	100				31.6				7	18	54	67	68	81		

Trimeth/Sulpha = Trimethoprim / Sulphamethoxazole; STA + = maltose positive *S. aureus*; STA - = maltose negative *S. aureus*; n = number of isolates

4 **Table S2.** Minimum inhibitory concentration (MIC) results of maltose positive and maltose negative *S. aureus* resistant to more than one antibiotic.

Isolate	(n)		Products																										
	T	Class	AC	AM	AZ	CE	CT	FX	CX	CP	DA	DP	ET	E	FX	FA	G	IM	LZ	ME	MU	OX	P	RI	SY	TEI	TE	TO	VA
STA-	2	1	S	R	S	S	S	S	S*	S	S	S	N/A	S	S	S	S	S*	S	S*	S	S	R	S	S	S	S	S	S
STA+	2	1	S	R	S	S	S	S	S	S	S	S	S	S	S	S	R	S	S	S	S	S	S	S	S	S	S	S	S
STA-	2	1	S	R	S	S	S	S	S	S	S	S	S	S	S	S	S	S	S	S	S	S	S	S	S	S	R	S	S
STA-	2	1	S	R	S	S	S	S	S	S	R	S	S	S	S	S	S	S	S	S	S	S	S	S	S	S	S	S	S
STA-	2	1	S	R	S	S	S	S	S	S	I	S	S	S	R	S	S	S	S	S	S	S	S	S	S	S	S	S	S
STA-	2	1	S	R	S	S	S	S	S	S	S	S	S	S	S	S	S	S	S	S	S	S	S	S	S	S	S	S	R
STA-	3	3	S	R	S	S	S	S	S	S	S	S	S	R	S	S	S	S	S	S	S	S	S	S	S	S	S	S	R
STA-	3	2	S	R	S	S	S	S	S	S	S	S	N/A	S	S	S	R	S	S	S	S	S	R	S	S	S	S	S	S
STA-	3	2	S	R	S	S	S	S	S	R	S	S	N/A	S	S	S	S	S	S	S	S	S	R	S	S	S	S	S	S
STA-	3	2	S	R	S	S	S	S	S	S	S	S	S	S	R	S	S	S	S	S	S	S	R	S	S	S	S	S	S
STA+	3	2	S	R	S	S	S	S	S	R	S	S	S	S	S	S	S	S	S	S	S	S	R	S	S	S	S	S	S
STA+	3	2	S	R	S	S	S	S	S	S	S	S	S	S	S	S	S	S	S	S	S	S	R	S	S	S	R	S	S
STA-	4	3	S	R	S	S	S	S	S	S	S	S	S	S	S	S	R	S	S	S	S	S	S	S	R	S	S	S	R
STA-	3	3	S	R	S	S	S	S	S	S	R	S	S	S	S	S	S	S	S	S	S	S	S	S	R	S	S	S	R
STA-	3	3	S	R	S	S	S	S	S	S	R	S	S	S	S	S	S	S	S	S	S	S	S	S	S	S	R	S	S
STA-	3	3	S	R	S	S	S	S	S	S	R	S	S	R	S	S	S	S	S	S	S	S	S	S	S	S	S	S	S
STA+	4	3	S	R	R	S	S	S	S	S	R	S	S	R	S	S	S	S	S	S	S	S	S	S	S	S	S	S	S
STA-	4	3	S	R	S	S	S	S	S	S	I	S	S	S	S	S	R	S	S	S	S	S	R	S	I	S	S	S	R
STA-	4	3	S	R	S	S	S	S	S	S	R	R	S	S	S	S	S	S	S	S	S	S	S	S	S	R	S	S	S
STA-	5	3	S	R	S	S	S	S	S	S	S	S	N/A	S	S	S	R	S*	S	S*	S	S	R	S	S	S	S	R	R
STA+	5	3	S	R	S	S	S	S	S	R	S	S	S	S	S	S	R	S	S	S	S	S	R	S	S	S	S	R	S
STA+	5	2	R	R	S	S	S	S	S	S	I	S	R	S	S	S	S	R	S	S	S	S	R	S	S	S	S	S	S
STA-	5	3	S	R	S	S	S	S	S	S	S	S	S	S	S	S	R	S	S	S	S	S	S	R	R	S	S	S	R
STA-	5	3	S	R	S	R	R	S	S	S	R	S	S	S	R	S	S	S	S	S	N/A	S	S	S	S	S	S	S	S
STA-	4	3	S	R	S	S	S	S	S	S	S	S	N/A	S	R	S	S	S*	S	S	S	S	R	R	S	S	S	S	S
STA-	5	3	S	R	S	S	S	S	S	S	S	S	N/A	S	S	S	R	S*	S	S	S	S	R	S	S	S	S	R	R
STA-	6	3	S	R	S	S	S	S	S	S	R	R	S	R	S	S	S	S	R	S	S	S	S	S	R	S	S	S	S
STA-	9	3	R	R	S	R	R	S	R	S	S	S	N/A	S	S	S	S	R	S	R	S	R	R	S	S	S	S	S	S
STA-	12	3	S	R	S	S	S	S	S	S	R	R	R	R	R	S	S	S	R	S	R	S	R	S	R	R	S	S	R
STA-	14	3	R	R	S	R	R	S	R	S	I	R	N/A	S	S	S	S	R	R	R	S	R	R	S	R	S	S	S	R
STA+	16	3	R	R	R	R	R	R	R	S	R	S	R	S	S	S	R	R	S	R	N/A	R	R	S	R	S	S	R	S
STA-	16	3	R	R	S	R	R	S	R	S	R	R	N/A	I	S	R	S	R	R	R	N/A	R	R	S	R	R	S	S	R
STA-	18	3	R	R	S	R	R	S	R	S	R	R	N/A	R	S	R	S	R	R	R	R	R	R	S	R	R	S	S	R
STA-	20	3	R	R	R	R	R	S	R	S	R	R	N/A	R	S	R	S	R	R	R	N/A	R	R	R	R	R	R	S	R
STA-	21	3	R	R	S	R	R	S	R	S	R	R	N/A	R	R	R	R	R	R	R	R	R	R	S	R	R	S	S	R
STA-	20	3	R	R	S	R	R	S	R	S	R	R	N/A	R	R	R	R	R	R	R	R	R	R	S	R	R	R	S	R
STA-	21	3	R	R	S	R	R	S	R	S	R	R	N/A	R	R	R	R	R	R	R	R	R	R	S	R	R	S	R	R

T = Total number of antimicrobial products resistant, Class = Number of antimicrobial classes that each isolate is resistant to, S = Susceptible, R = Resistant, I = Intermediate, N/A = Not applicable, (n) = number of isolates, AC = Amox/K Clav, AM = Ampicillin, AZ = Azithromycin, CE = Cefepime, CT = Cefotaxime, FX = Cefoxitin Screen, CX = Cefuroxime, CP = Ciprofloxacin, DA = Clindamycin, ET = Ertapenem, E = Erythromycin, FO = Fosfomycin, FA = Fusidic Acid, G = Gentamycin, IM = Imipenem, LZ = Linezolid, ME = Meropenem, MU = Muriprocin, OX = Oxacillin, P = Penicillin, RI = Rifampin, SY = /Synercid (Quinupristin-Daldopristin), TEI = Teicoplanin, TE = Tetracycline, TO = Tobramycin, VA = Vancomycin
